# Older Adults’ Intention to Socially Isolate Once COVID-19 Stay-at-Home Orders Are Replaced With “Safer-at-Home” Public Health Advisories: A Survey of Respondents in Maryland

**DOI:** 10.1177/0733464820944704

**Published:** 2020-07-22

**Authors:** Michael A. Callow, Daniel D. Callow, Charles Smith

**Affiliations:** 1 Morgan State University, Baltimore, MD, USA; 2 University of Maryland, College Park, USA; 3 University of Maryland, Baltimore, USA

**Keywords:** risk perception, politics, policy, health behaviors, COVID-19, social distancing, Theory of Planned Behavior

## Abstract

**Background:** The Theory of Planned Behavior (TPB) and the Health Belief Model (HBM) were used to examine the opinion and behaviors of older adults regarding Coronavirus Disease 2019 (COVID-19), social distancing practices, stay-at-home orders, and hypothetical public policy messaging strategies. **Method:** A convenience sample (*N* = 242) of adults 60 and older in the state of Maryland took part in an online survey. Respondents filled out questions regarding demographic information, political affiliation, current social distancing behaviors, and TPB and HBM constructs in our proposed model. Linear regression analysis and analysis of covariance (ANCOVA) were conducted to test the model. **Results:** Attitude toward social isolation was affected by perceived benefits and barriers to social distancing measures, perceived severity of COVID-19, and political affiliation. Behavior intention was influenced by attitude, subjective norms, political affiliation, and messaging strategies. **Conclusion:** The study provides support for the conceptual model and has public policy implications as authorities begin to lift stay-at-home orders.

## Introduction

The global response to the Coronavirus Disease 2019 (COVID-19) pandemic has led to dramatic changes to human behavior as public officials weigh the health of their citizens and the impact on the economy with stay-at-home and social distancing orders (Lin & Meissner, 2020). Researchers have carried out extensive analyses to provide accurate estimates of the case fatality rate of COVID-19 (Verity et al., 2020) as new data across various countries emerge. An underlying trend is a significant correlation between age and case fatality ratios; a study published in March 2020 estimated the fatality ratio for reported COVID-19 cases in the United States to be around 1.8% to 3.4% overall (CDC COVID-19 Response Team, 2020). This number was as low as 0.1% to 0.2% among those aged 20 to 44, increasing to 0.5% to 0.8% among those aged 45 to 54, 1.4% to 2.6% among 55 to 64, 2.7% to 4.9% among 65 to 74, 4.3% to 10.5% among 75 to 84, and 10.4% to 27.3% among those aged 85 and older. In addition, the percentage of individuals requiring hospitalization and intensive care unit (ICU) admission from complications arising from COVID-19 increases with age. With the relative success of stay-at-home and social distancing orders in “flattening the curve” leading to more states lifting these restrictions and opening up the economy, the health risks of COVID-19 remain for seniors.

The balancing act between reopening the economy and protecting public health has become highly politicized throughout the nation. A lack of a consistent and clear message regarding COVID-19 at the national level has led to divergent opinions as to the severity of the virus across political ideologies (Singal, 2020). A case in point is the wearing of face masks in public places. In early March, the World Health Organization (WHO) and the Centers for Disease Control and Prevention (CDC) stated that the general public should not purchase or wear face masks, in part because they were worried that it would lead to a shortage of masks for essential health care workers (Aratani, 2020; Yan, 2020). By early April, the U.S. Surgeon General and the CDC reversed this guidance, urging people to wear a cloth face mask to protect others from getting infected from asymptomatic carriers of COVID-19. By late June, the White House coronavirus task force coordinator stated that there was some scientific evidence to suggest that wearing a mask could also lower the risk of getting infected. This confusing public messaging may have contributed to a “culture war” within the general public with regard to wearing masks in public; those who refuse to wear masks claim that it goes against their individual freedom and constitutional rights (Aratani, 2020).

In the state of Maryland, a “stay-at-home” order was put into effect on March 20, 2020. Residents were ordered to stay in their homes or places of residence except to conduct essential activities (such as food shopping or visiting the doctor) or permitted outdoor activities (exercising). Meanwhile, nonessential businesses were ordered to close, including senior centers. On May 13, the Governor of Maryland announced that the state would move to Phase One of a three-part phase for lifting restrictions on Marylanders. Still, he noted that residents need to continue social distancing protocols for this first stage of the recovery plan to be successful (CBS Baltimore, 2020). Although the state of Maryland officially transitioned from a stay-at-home order to a safer-at-home public health advisory on May 15 at 5:00 p.m., it empowered local authorities to make decisions regarding the timing of reopening businesses (The Office of Governor Larry Hogan, n.d.).

A potential public policy initiative as communities begin to lift restrictions for the wider population is to provide advisories or mandates for continued social distancing protocols (referred to as “shielding” in the United Kingdom) among those populations who are at greater risk from COVID-19. Indeed, the policy of requiring the younger population to maintain social distancing is, in large part, to reduce infection rates in high-risk populations such as the elderly. As political and economic pressures mount on authorities to lift restrictions, public policy initiatives may shift more to targeting those at higher risk with stay-at-home and social distancing orders as opposed to more generalized social distancing initiatives. Indeed, in England, the government recommended that some 2.2 million individuals deemed to be clinically extremely vulnerable from COVID-19 to “shield” (similar to shelter-in-place), essentially avoiding all contact from others as much as possible (Davis, 2020). However, health experts have expressed concern that extended social isolation among older adults may lead to a greater risk of cardiovascular, autoimmune, neurocognitive, and mental health problems (Armitage & Nellums, 2020; Brooke & Jackson, 2020). Government and public health officials, therefore, must consider the most effective measures for protecting older adults from COVID-19 while recognizing the potential risks that social isolation orders may have on their mental and physical health.

The purpose of this study is to examine the opinion and social distancing behaviors of older adults and to explore how they react to hypothetical public policy interventions targeted toward their age group as the rest of the population eases back into less restricted stay-at-home orders. In particular, we gauged the attitude toward social distancing orders of older adults in the state of Maryland in the week leading up to the Governor’s lifting of restrictions on May 15, 2020. We also examined respondents’ reactions toward different public policy interventions targeted toward older adults to determine which messaging strategies elicited higher intentions to maintain social distancing behaviors moving forward. Given the politicization of the pandemic, we also compared responses based on political beliefs.

### Conceptual Framework

The Theory of Planned Behavior (TPB) and the Health Belief Model (HBM) were used to develop a model for helping to predict social distancing behavior. The TPB, in particular, has been widely used as a framework for predicting health behaviors (Godin et al., 2010). A fundamental tenet is that behavior intention is the best predictor of actual behavior that requires some degree of self-control (Ajzen, 2002). The TPB argues that behavior intention is greater when the person’s *attitude* toward the behavior is favorable, when others who are important to the individual approve of that behavior (*subjective norm*), and when the person believes that they are capable of performing the behavior (*perceived behavioral control*). In this instance, the behavior in question is social isolation behavior such as staying at home, practicing social distancing, and wearing a mask when in public places.

The HBM has also been used extensively in the public health literature (Carpenter, 2010; C. J. Jones et al., 2014; C. L. Jones et al., 2015; Krawczyk et al., 2012) and has been used for more recent research into COVID-19 (Carico et al., 2020; Mukhtar, 2020). This model states that people are more likely to adopt a health behavior (i.e., stay at home) if they believe that they are at high risk of being infected (*perceived susceptibility*), the disease poses significant health risks (*perceived severity*), there are substantial *benefits* and few *barriers* from engaging in the behavior, and they are exposed to *cues to action* that are persuasive (Rosenstock, 1974). These cues to action can be in the form of carrots or sticks to promote public health behaviors (Blacksher, 2008; Rothschild, 1999). Carrots are essentially monetary or nonmonetary rewards for adopting the desired behavior, whereas sticks are monetary or nonmonetary punishments for adopting the competing behavior (Lee & Kotler, 2019).

Political affiliation is also expected to have an effect on an individual’s attitude toward social isolation. Previous studies have shown that political affiliation can have an impact on public health initiatives (Baum, 2011). As noted previously, inconsistent public policy messaging at the national level has led to the politicization of COVID-19, with conservatives tending to downplay the severity of the virus when compared with liberals (Aratani, 2020). We therefore predict that public health messaging strategies will be moderated by political affiliation depending on whether respondents believe the policy reflects “the greater good” or “government overreach.”

**Figure 1.**
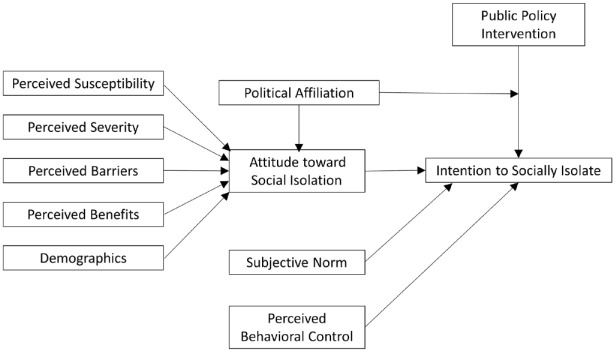
Conceptual model for intention to socially isolate.

Figure 1 outlines our proposed conceptual model for predicting older adults’ intention to maintain social distancing practices once stay-at-home restrictions are lifted for the general population. Similar to the study by Krawczyk et al. (2012), it incorporates the concepts from TPB and HBM.

Older adults’ intention to socially isolate and maintain social distancing behaviors when the community transitions from a “stay-at-home” order to a “safer-at-home” public health advisory is expected to be directly influenced by their overall attitude toward social isolation and social distancing practices, by the opinion of influential others (friends and family), and by perceived behavioral control. In addition, their overall attitude toward this behavior is determined by the perceived susceptibility to and severity of COVID-19, as well as the perceived benefits and barriers of social distancing and social isolation practices. Political affiliation along with various demographic variables, such as age, gender, race, income, and education level, were also included to see whether they have an impact on a person’s attitude toward social isolation. Finally, various public policy messages (carrots vs. sticks) were used as cues to action to determine whether they had a positive or negative impact on behavior intention compared with a control group. Political affiliation is hypothesized to have a moderating effect on how these cues affect behavior intention, with more democratic respondents favoring government initiatives put in place to protect those at risk versus more Republican respondents perceiving these initiatives to be unnecessary.

## Method

### Study Design and Participants

“The COVID-19 Outbreak, Social Distancing, and Mary-land’s Stay-at-Home Order” survey is a descriptive cross-sectional study that drew from a convenience sample of adults over the age of 60 in the state of Maryland, between May 12 and May 15, 2020, leading up to the transition from a stay-at-home order to a safer-at-home public health advisory on May 15, at 5:00 p.m.

### Survey

An online Qualtrics survey of 60 questions (estimated to take under 10 min to complete) was used to collect data. The survey was approved by the Morgan State University Institutional Review Board, and informed consent was obtained from all participants prior to proceeding with the survey. The survey included questions regarding demographic information, political affiliation, current social distancing behaviors, exercising behaviors, general anxiety, and TPB and HBM constructs in our proposed model. Social media advertising was used as the primary method of recruitment to reach the targeted population (age 60+) living in Maryland. Advertising time was purchased on Facebook, because this is the preferred social media for U.S. adults aged 65+ and is accessed by most on a daily basis (Perrin & Anderson, 2019). Approximately 10,600 individuals were reached by the Facebook advertisement over 3 days, 341 of whom engaged with the advertisement (reaching the informed consent page with the survey link and starting the survey) and 245 individuals completed the survey (the survey did not allow respondents to skip questions). We excluded three participants who indicated that they were younger than 60, leaving us with a sample size of 242.

### Measures

Participants were asked to provide demographic information, including age, sex, race/ethnicity, income, education level, employment status, household size, and zip code. Respondents were also asked whether they or any household member had tested positive for COVID-19. They were asked various questions regarding their current social distancing behavior (whether they were following official guidelines in their area, the reasons why they leave home, and whether they wear a protective mask in public places), their level of physical activity, and their political affiliation. Respondents were also assessed for general anxiety using the Geriatric Anxiety Scale–10 (GAS-10) Item Version (Mueller et al., 2015).

Measures of the constructs from HBM and TPB were adapted from scales used by Krawczyk et al. (2012) and Champion (1999). Each item was measured using a 7-point Likert-type scale unless otherwise indicated. Two items were used to measure the benefits of social isolation (*will keep me safe from COVID-19; will help diminish the spread of COVID-19*). Six items were used to measure barriers to staying at home (*other problems that are more important; too painful; too difficult; not good for my physical health; not good for my mental health; worries me because it will harm the economy*). One item was used to measure perceived susceptibility (*it is likely that I will get COVID-19 in the near future*), and another item was used to measure perceived severity (*I think that if I were to get COVID-19, it would be serious for my health*). Attitude was measured using two items (*I believe staying at home is a good idea; I would recommend that people stay at home*). Subjective norms were measured using two items (*most people who are important to me think that I should stay at home; it is expected of me to stay at home*). Perceived behavioral control was measured using one item (*deciding to stay at home is beyond my control*, reverse coded).

For the intervention, all participants were presented with a scenario that the state was moving forward with a new phase in opening up the economy while maintaining various social distancing practices when in public. They were also told that even though the risk of catching COVID-19 has decreased, there is still no cure for the disease and that there is a possibility of a second pandemic until a vaccine is widely available. Respondents were then randomly assigned to one of the following conditions: (a) *Control*: Stay-at-home order has been lifted for all Maryland residents; (b) *Mandatory Enforced Intervention*: Stay-at-home order remains in effect for Maryland residents aged 65+ as well as people of any age who have serious underlying medical conditions. Offenders may be subject to imprisonment not exceeding 1 year or a fine not exceeding US$5,000 or both; (c) *Mandatory Unenforced Intervention*: Stay-at-home order remains in effect for Maryland residents aged 65+ as well as people of any age who have serious underlying medical conditions. The order will not be enforced by state and local law enforcement, and there is no penalty for residents 65 and older who choose to ignore this directive; (d) *Safer-at-home*: The stay-at-home order has been lifted for all Maryland residents. However, state public health officials and state leaders recommend that residents aged 65+, as well as people of any age who have serious underlying medical conditions, continue to stay at home as much as possible for their own safety; (e) *The Facts*: The stay-at-home order has been lifted for all Maryland residents. Respondents were then provided with data from the CDC on fatality rates by age group and were advised that there was still a risk that residents could still be infected by COVID-19, which could result in severe disease, including hospitalization, admission to an ICU, and death, especially among older adults.

Behavior intention was measured using three items (*I intend to stay at home in isolation as much as possible; I intend to follow guidelines in my area on social distancing; I intend to wear a protective mask when I have to interact with others outside my house, that is, when shopping or going to the doctor’s office or pharmacy*).

## Statistical Analyses

The conceptual framework presented above was used to guide all analyses. A multiple linear regression analysis was used to examine the impact of HBM variables on ATTITUDE. Pearson’s correlations were conducted on the independent variables, including descriptives. Intercorrelations were generally low (*r* < .4), although moderate correlations were found between BENEFITS and SERIOUSNESS (*r* = .540, *p* < .01) and BENEFITS and BARRIERS (*r* = −.475, *p* < .001). Issues of multicollinearity were initially checked by calculating variance inflation factors (VIFs) for all predictive variables in the model, with BENEFITS reporting the highest value of 2.06, well below the recommended cutoff value of 5 (Vatcheva & Lee, 2016). All conceptual variables were included in the regression, along with an array of demographic variables and political affiliation.

To assess respondents’ intention to socially isolate once the general stay-at-home order was lifted, an analysis of covariance (ANCOVA) was run to compare the five groups (control and four interventions), including the TPB variables as covariates. Levene’s test for equivalence was run to check for homogeneity, with a *p* value of less than .01. However, analysis of variance is reasonably robust to violations of this assumption if the sizes in the groups are reasonably similar (Box, 1953; Stevens, 2001). In this instance, counts ranged from 47 to 49 across the five groups and are therefore almost equal. ANCOVA was therefore used to assess factors that affect behavior intention. All analyses were conducted using the statistical software program JASP (JASP Team, 2020).

## Results

A total of 242 completed survey responses were included in the analyses. Table 1 provides an overview of respondents’ demographic profile, political affiliation, and level of anxiety. In terms of age distribution, the largest age groups were 70- to 79-year-olds (36.8%), followed by 65- to 69-year-olds (34.7%) and 60- to 64-year-olds (22.7%). A significant majority of respondents were female (76.9%). The majority had received either an undergraduate degree (28.1%) or a graduate degree (23.2%), with 28.7% having started but not completed a college degree. A significant majority of the respondents were retired (65.0%), 28.1% were employed or self-employed, and 2.1% were unemployed. Most of the respondents were White (88.8%), with 4.1% identifying themselves as Black or African American; and 57.0% lived with one other person, 20.7% lived by themselves, and 14.2% reported that they lived with two other people.

**Table 1.** Descriptive Information of Survey Respondents.

Descriptive variable	Total sample (*N* = 242) *n* (%)
Demographics	
Age	
60–64	59 (24.3%)
65–69	84 (34.7%)
70–79	89 (36.8%)
80–89	10 (4.1%)
90+	0
Sex	
Male	56 (23.1%)
Female	186 (76.9%)
Race	
White	215 (88.8%)
Black	10 (4.1%)
Hispanic	1 (0.4%)
Asian	3 (1.2%)
American Indian/Alaskan/Native Hawaiian/Pacific Islander	4 (1.6%)
More than one	2 (0.8%)
Prefer not to say	7 (2.9%)
Education	
Some high school	2 (0.8%)
High school diploma/GED	18 (7.4%)
Some college/associate degree/vocational degree	68 (28.1%)
College degree	69 (28.5%)
Graduate degree	57 (23.6%)
Doctoral degree	28 (11.6%)
Employment	
Retired	159 (65.7%)
Employed	50 (20.7%)
Self-employed	17 (7%)
Unemployed	5 (2.1%)
Other	11 (4.5%)
Political affiliation	
Strong Democrat	68 (28%)
Moderate Democrat	55 (22.7%)
Independent	40 (16.5%)
Moderate Republican	48 (19.8%)
Strong Republican	28 (11.6%)
Completely uninvolved	3 (1.2%)
GAS-10	
Minimal anxiety	136 (56.2%)
Mild anxiety	59 (24.4%)
Moderate anxiety	26 (10.7%)
Severe anxiety	21 (8.7%)

*Note.* GED = General Educational Development; GAS-10 = Geriatric Anxiety Scale–10 Item Version.

Based on the GAS-10, 24.4% were minimally anxious, 56.2% were mildly anxious, 10.7% were moderately anxious, and 8.9% would be considered severely anxious. Maryland is primarily known as a “blue” state. Just over half of the respondents (50.8%) identified as Democrat and 31.4% identified as Republican.

For reliability analysis, Pearson’s *r* was used for scales with two items and Cronbach’s alpha was used for scales with three or more items. The reliability for benefits (*r* = .733, *p* < .01), barriers (σ = 0.820), attitude (*r* = .877, *p* < .01), subjective norms (*r* = .792, *p* < .01), and behavior intention (σ = .838) were acceptable, and individual constructs were created for each scale by summing up the scores for the scale items.

To test for the influence of HBM variables on attitude toward social isolation, a multiple linear regression analysis was conducted with attitude regressed on HBM variables, demographic factors, and political affiliation (see Table 2). The adjusted *R*^2^ was .747, suggesting the model explained 74.7% of the variance in attitude and was a good fit. It should be noted that low attitudinal scores suggest strong agreement (positive attitude) with social isolation, whereas higher scores reflect strong disagreement (negative attitude) with social isolation. The mean value for attitude among the entire sample was 4.41, ranging from 2 to 14.

**Table 2.** Multiple Regression—ATTITUDE.

Predictors		*b*	*SE*	β	*t*	*p*
(Intercept)		2.014	1.187		1.697	.091
Age		−0.084	0.120	−.025	−0.700	.485
Gender		−0.102	0.240	−.014	−0.423	.672
Employment		0.073	0.083	.031	0.879	.380
Education		0.032	0.094	.012	0.343	.732
Political affiliation		0.377	0.119	.131	3.164	.002
Benefits		0.648	0.053	.568	12.199	<.001
Barriers		−0.095	0.016	−.228	−5.766	<.001
Susceptible		0.136	0.076	.060	1.782	.076
Serious		0.263	0.081	.128	3.251	.001
Model	*R*²	Adjusted *R*²	*R*² Change	*F* Change	*df*	*p*
H_0_	.000	.000	.000		(0, 241) (9, 232)	
H₁	.757	.747	.757	80.099		<.001

*Note.* Nonstandardized (*b*) and standardized beta (β) coefficients, along with standard errors, are reported.

The nonstandardized (*b*) and standardized beta (β) coefficients for all variables in the multiple linear regression are reported in Table 2. Age, gender, income, and education level were not statistically significant, which may be due to the relative homogeneity across these variables within the sample. Political affiliation, on the contrary, did have a statistically significant effect on ATTITUDE (β = .131, *p* < .01). As shown in Table 3, a clear pattern emerges, with those individuals identifying as strong democrats displaying the lowest mean values (*M* = 2.603, *SD* = 0.995) and, therefore, more positive attitudes toward social isolation practices. In contrast, strong Republicans achieved the highest mean values (*M* = 8.071, *SD* = 4.018), representing a lower attitudinal disposition toward social isolation practices.

**Table 3.** Attitude Toward Social Isolation by Political Affiliation.

Political affiliation	*M*	*SD*	*n*
Strong Democrat	2.603	0.995	68
Moderate Democrat	3.345	1.554	55
Independent	4.450	2.801	40
Moderate Republican	6.104	3.217	48
Strong Republican	8.071	4.018	28

*Note.* Attitude Toward Social Isolation Scale: 2 = *highly favorable*, 14 = *highly unfavorable*.

Table 4 provides descriptive statistics for the HBM variables that were predicted to have an impact on attitude toward social isolation. In each case, lower values reflect agreement with the concept and higher values reflect disagreement. As expected, the variable BARRIERS had a statistically significant effect on ATTITUDE (β = −.228, *p* < .01), as did BENEFITS (β = .568, *p* < .01). There was also directional support because the belief that there were significant barriers to social distancing practices had a lowering effect on attitude and the belief that there were significant benefits had a positive effect on attitude. Respondents who believed that COVID-19 was a serious threat (β = .128, *p* < .01) tended to exhibit a greater positive attitude toward social distancing practices. The variable SUSCEPTIBLE was not statistically significant (*p* > .05).

**Table 4.** Descriptive Statistics for Attitude and Health Belief Model Variables.

	Attitude	Barriers	Benefits	Serious	Susceptible
Valid	242	242	242	242	242
*M*	4.409	28.058	4.562	2.326	4.364
*SD*	3.042	7.331	2.663	1.487	1.348
Minimum	2.000	6.000	2.000	1.000	1.000
Maximum	14.000	42.000	14.000	7.000	7.000

An ANCOVA was conducted to test the effects of different types of public policy messages on respondents’ intention to continue to socially isolate. As expected from TPB, attitude and subjective norms have an impact on behavior intention (*p* < .001). However, perceived behavioral control was not statistically significant. The public policy messages were statistically significant (*p* < .05), as was political affiliation (*p* < .001). However, the interaction between the interventions and political affiliation was not statistically significant (*p* > .05). This may be because the cell sizes were particularly small, given sample size, ranging between four and 16 respondents (see Table 6). Effect sizes were estimated using partial eta squared ($\eta_{\text{p}}^{2}$). Large effect sizes were recorded for political affiliation ($\eta_{p}^{2}$ = 0.498) and attitude ($\eta_{\text{p}}^{2}$ = 0.587), while subjective norms provided a medium effect size ($\eta_{p}^{2}$ = 0.103) and the intervention cues provided a small effect size ($\eta_{p}^{2}$ = 0.047). Table 5 provides an overview of the ANCOVA results and effect sizes, and Table 6 identifies the mean values for each intervention across political affiliations.

**Table 5.** ANCOVA—Behavior Intention.

Cases	Sum of squares	*df*	Mean square	*F*	*p* value	$\eta_{p}^{2}$
PPM	31.09	4	7.77	2.462	.046	0.047
PA	48.27	4	12.07	3.821	.005	0.498
PPM × PA	65.21	16	4.08	1.290	.205	0.089
Perceived behavioral control	1.56	1	1.56	0.493	.483	0.001
Attitude	377.15	1	377.15	119.432	<.001	0.587
Subjective norms	68.06	1	68.06	21.554	<.001	0.103
Residuals	666.30	211	3.2			

*Note.* Type III sum of squares. $\eta_{p}^{2}$ indicates partial eta squared. ANCOVA = analysis of covariance; *df* = degrees of freedom; PPM = public policy messaging; PA = political affiliation.

**Table 6.** Descriptives—Behavior Intention.

Public policy messages	Political affiliation	*M*	*SD*	*n*
Control	Strong Democrat	4.333	1.371	12
	Moderate Democrat	4.636	1.362	11
	Independent	6.857	4.298	7
	Moderate Republican	6.833	2.082	12
	Strong Republican	8.000	3.795	6
	Total	5.816	2.751	48
Mandatory and enforced	Strong Democrat	4.077	1.188	13
	Moderate Democrat	4.625	1.061	8
	Independent	6.300	2.214	10
	Moderate Republican	6.364	2.942	11
	Strong Republican	11.167	5.879	6
	Total	6.041	3.397	48
Mandatory but unenforced	Strong Democrat	3.750	0.856	16
	Moderate Democrat	4.750	1.485	12
	Independent	6.333	4.227	6
	Moderate Republican	6.500	2.976	8
	Strong Republican	7.167	4.792	6
	Total	5.208	2.851	48
Safer-at-home advisory	Strong Democrat	3.800	1.146	15
	Moderate Democrat	4.214	1.051	14
	Independent	6.889	3.516	9
	Moderate Republican	4.800	2.490	5
	Strong Republican	9.500	7.176	6
	Total	5.286	3.518	49
Scientific facts	Strong Democrat	3.750	1.055	12
	Moderate Democrat	5.000	1.826	10
	Independent	7.000	4.781	8
	Moderate Republican	7.333	2.309	12
	Strong Republican	10.000	5.888	4
	Total	6.021	3.404	46

*Note.* Behavior Intention Scale: 3 = *high intention*, 20 = *low intention*.

A post hoc analysis using Tukey’s *t*-test for behavior intention scores across political affiliation revealed statistically significant differences between individuals who identified as strong Republicans (*M* = 9.107, *SD* = 5.377) and those who identified as strong Democrats (*M* difference = −5.181, *t* = −8.382, *p* < .001), moderate Democrats (*M* difference = −4.489, *t* = −7.025, *p* < .001), Independents (*M* difference = −2.432, *t* = −2.432, *p* < .01), and moderate Republicans (*M* difference = −2.524, *t* = −3.856, *p* = .001). Overall, strong Republicans reported lower intentions to follow social distancing orders or advisories when compared with others. Those who identified as being strong Democrats, on the contrary, claimed to be more likely to follow social distancing orders/advisories (*M* = 3.926, *SD* = 1.111) when compared with Independents (*M* difference = 2.749, *t* = 5.011, *p* < .001) and moderate Republicans (*M* difference = 2.657, *t* = 5.120, *p* < .001). Moderate Democrats also claimed to be more likely to follow social distancing rules/advisories (*M* = 4.618, *SD* =1.354) compared with moderate Republicans (*M* difference = 1.965, *t* = 3.615, *p* < .01) and Independents (*M* difference = 2.057, *t* = 3.596, *p* < .01).

## Discussion

The main purpose of this study was to test a conceptual model that helps explain adult seniors’ attitudes toward social isolation and to determine their intention to follow social distancing policies once the community begins to lift restrictions to the general population. Older adults are an at-risk group for COVID-19, and government and public health officials will need to balance the desire to lift restrictions on low-risk and no-risk groups while protecting higher risk groups.

In developing our model, we borrowed two frameworks that have been used extensively in public health research: the HBM and the TPB. Our expanded model proposes that HBM variables can help shape a person’s attitude toward social isolation. In particular, we found that the perceived benefits of social distancing behaviors, along with the perceived seriousness of COVID-19 to one’s health, had a positive impact on attitude toward social distancing measures. At the same time, perceived barriers to social distancing measures had the expected negative impact on attitude toward social isolation. In our second analysis, we tested the TPB variables’ effect on older adults’ intention to practice social distancing in the near future once the community had begun to lift restrictions for certain businesses and groups of individuals. In conformance with Ajzen’s TPB model, attitude toward social isolation as well as the social influence of friends and family (subjective norms) played a big part in shaping older adults’ intention to remain socially distant or become less rigid in maintaining social distancing behaviors. However, perceived behavioral control (self-efficacy) had no impact on behavior intention.

The HBM includes cues to action as a determinant of behavior. In this article, we examined the use of messages reflecting either a carrot or a stick approach. In essence, the carrot approach focuses on the benefits of the desired social behavior, whereas the stick approach pursues barriers to the undesired behavior. Stay-at-home orders that are enforced by fines and potential imprisonment are likely to be perceived as enforcing the “big stick,” whereas safer-at-home public policy advisories tend to focus on promoting the safety of the public by outlining the benefits of remaining socially isolated. The mandatory enforced public messaging strategy tended to fare the worst with the lowest behavior intention (*M* = 6.041), along with the scientific facts intervention (*M* = 6.021). The mandatory but unenforced condition fared the best for behavior intention (*M* = 5.208) as well as the safer-at-home advisory (*M* = 5.286).

An additional interest of this article was the role that political affiliation may play in people’s attitude toward social distancing practices and their behavior intention given different messaging strategies. Political affiliation did seem to make a difference in terms of attitude toward social isolation practices. Individuals who identified themselves as being a strong Democrat had the most positive attitude toward social isolation (*M* = 2.603, *SD* = 0.995), whereas those who identified as being strong Republicans had the lowest average scores (*M* = 8.071, *SD* = 4.081). Although we predicted that political affiliation would moderate the effects of the public policy messaging strategies on behavior intention, the interaction effect was not statistically significant. This is perhaps due to the limited sample size. A cursory examination of the mean scores between political affiliations within and across the interventions in Table 6 provides some glimpses as to the role that political affiliation may play as a moderating role with seemingly different likes and dislikes across political affiliations for the various public policy messaging strategies.

### Study Limitations

The main limitation of this study is the sample size. A day before we were about to launch the survey, Maryland announced that it would begin to relax stay-at-home restrictions by the end of the week. We, therefore, had only three full days to collect survey responses online. Although the sample size (*n* = 242) was not of great concern for most of the analyses, the proposed interaction between the proposed intervention (five levels assigned randomly) and political affiliation (five categories, self-identified) meant that some cell sizes would be too small to uncover statistically significant results.

Respondents should also be wary of generalizing results to the state of Maryland, because the convenience sample was disproportionately female and White and was selected via social media. The data provided a good test of the model, but caution should be taken in assuming that these results are representative of a larger population or even, for that matter, a political affiliation. A larger, more representative sample would be needed to make any such generalizations.

Future research should look at different types of messaging strategies and frameworks that examine the impact on not only behavior intention but also maybe other antecedent variables such as perceived barriers and benefits, or perceived severity or susceptibility. The carrot and stick approach is a useful framework that can include monetary and nonmonetary punishments and rewards for public policymakers to use, but a marketing approach (what Michael Rothschild refers to as “promises”) may also be just as influential in convincing people to “do the right thing” (Rothschild, 1999). Given the politicization of the pandemic, it would also be interesting to examine what targeted public health communication strategies would be effective in increasing the adoption of a vaccine for COVID-19 assuming it becomes available in the future. Future research could also examine monetary versus nonmonetary incentives (carrots) versus disincentives (sticks) for increasing the adoption rate of a vaccine (e.g., offering a US$15 retail coupon as a monetary incentive for getting a vaccine).

## Conclusion

This article has examined the opinions and behaviors of older adults regarding COVID-19, social distancing practices, and stay-at-home orders. The focus has been on the state of Maryland, because each state in the United States has taken different approaches to tackling the pandemic. The proposed model was developed by merging concepts from two conceptual frameworks that have been used extensively in the field of public health. In addition, the article has attempted to examine the influence of political affiliation in people’s attitude toward the pandemic and the government’s response.

From a public policy perspective, our proposed conceptual model provides government and health officials with an insight into how an individual’s beliefs, attitudes, and social influences affect intentions to self-isolate while there is no cure or vaccine for the disease. These officials have various means for influencing social distancing measures for the good of the individual and society. Knowing that the perceived benefits and barriers to social distancing measures, along with the perceived severity of COVID-19, have a significant impact on attitude toward social isolation can provide clues in terms of additional messaging strategies that alter these beliefs in a positive way. Political affiliation seems to also play a significant role in not only attitude but also behavior intention. This comes as no surprise, because the pandemic has become highly politicized over the last few months. This has been due in part to inconsistent public policy messaging at the national level, particularly with regard to the severity of the virus and the best practices for mitigating the spread of COVID-19 in neighborhoods. With the reopening of economies and a resurgence of cases of COVID-19 in local communities, officials may need to provide more targeted messaging to individuals from different political affiliations. Officials may want to consider the pros and cons of the carrot versus the stick approach to public policy orders and advisories. Using political spokespersons who are perceived to be more acceptable and credible to different targeted audiences may also help change people’s attitudes and behavior intentions.
